# A Style Transfer-Based Fast Image Quality Assessment Method for Image Sensors

**DOI:** 10.3390/s25165121

**Published:** 2025-08-18

**Authors:** Weizhi Xian, Bin Chen, Jielu Yan, Xuekai Wei, Kunyin Guo, Bin Fang, Mingliang Zhou

**Affiliations:** 1 Chongqing Research Institute of Harbin Institute of Technology, Harbin Institute of Technology, Chongqing 401151, China; 26974740@alu.cqu.edu.cn; 2 Faculty of Computing, Harbin Institute of Technology, Harbin 150001, China; 3 International Research Institute for Artificial Intelligence, Harbin Institute of Technology, Shenzhen 518055, China; 4 School of Computer Science, Chongqing University, Chongqing 400044, China; xuekaiwei2-c@my.cityu.edu.hk (X.W.); fb@cqu.edu.cn (B.F.); mingliangzhou@cqu.edu.cn (M.Z.); 5 National Elite Institute of Engineering, Chongqing University, Chongqing 400044, China; css@cqu.edu.cn

**Keywords:** image quality assessment, full reference, style transfer, image content, perceptual distance

## Abstract

Accurate image quality evaluation is essential for optimizing sensor performance and enhancing the fidelity of visual data. The concept of “image style” encompasses the overall visual characteristics of an image, including elements such as colors, textures, shapes, lines, strokes, and other visual components. In this paper, we propose a novel full-reference image quality assessment (FR-IQA) method that leverages the principles of style transfer, which we call style- and content-based IQA (SCIQA). Our approach consists of three main steps. First, we employ a deep convolutional neural network (CNN) to decompose and represent images in the deep domain, capturing both low-level and high-level features. Second, we define a comprehensive deep perceptual distance metric between two images, taking into account both image content and style. This metric combines traditional content-based measures with style-based measures inspired by recent advances in neural style transfer. Finally, we formulate a perceptual optimization problem to determine the optimal parameters for the SCIQA model, which we solve via a convex optimization approach. Experimental results across multiple benchmark datasets (LIVE, CSIQ, TID2013, KADID-10k, and PIPAL) demonstrate that SCIQA outperforms state-of-the-art FR-IQA methods. Specifically, SCIQA achieves Pearson linear correlation coefficients (PLCC) of 0.956, 0.941, and 0.895 on the LIVE, CSIQ, and TID2013 datasets, respectively, outperforming traditional methods such as SSIM (PLCC: 0.847, 0.852, 0.665) and deep learning-based methods such as DISTS (PLCC: 0.924, 0.919, 0.855). The proposed method also demonstrates robust generalizability on the large-scale PIPAL dataset, achieving an SROCC of 0.702. Furthermore, SCIQA exhibits strong interpretability, exceptional prediction accuracy, and low computational complexity, making it a practical tool for real-world applications.

## 1. Introduction

Accurate image quality evaluation is essential for optimizing sensor performance and enhancing the fidelity of visual data [1,2,3]. Digital imaging systems are integral to modern technological advancements and play pivotal roles in diverse applications, such as surveillance [4], video coding [5,6], video enhancement [7,8], diagnostics [9], autonomous systems [10], and environmental monitoring [11,12]. However, the acquisition, processing, compression, transmission, and display of digital images are prone to various distortions that significantly affect image fidelity. These distortions not only compromise the accuracy of visual information but also degrade the overall performance of imaging systems, leading to reduced reliability and user satisfaction. Therefore, the development of robust image quality assessment (IQA) methodologies has become a critical area of research in sensor technology and computer vision.

The practical significance of IQA is evident across numerous real-world applications. In medical imaging, for example, accurate image quality assessment is crucial for ensuring the reliability of diagnostic tools, where even minor distortions can lead to misdiagnosis or delayed treatment [13]. In autonomous systems, such as self-driving cars, high-fidelity image quality is essential for reliable object detection and scene understanding [14], directly impacting safety and decision-making [15,16]. Similarly, in satellite and aerial imaging, IQA plays a vital role in maintaining the integrity of geospatial data, which is critical for environmental monitoring, urban planning, and disaster response [17,18,19]. These applications underscore the importance of developing robust, efficient, and interpretable IQA methods that can handle the complexities of real-world visual data.

Despite its importance, IQA faces several challenges in real-world scenarios. First, the diversity of image content and distortion types poses a significant challenge for traditional IQA methods, which often struggle to generalize across different domains and distortion severities [20]. Second, the increasing demand for real-time processing in applications such as video streaming and autonomous systems necessitates efficient IQA methods that can operate within stringent computational constraints [21]. Third, the interpretability of IQA results is often overlooked, making it difficult to diagnose the root causes of image degradation and improve system performance [22]. Addressing these challenges requires a holistic approach that balances perceptual accuracy, computational efficiency, and interpretability.

Full-reference image quality assessment (FR-IQA) serves as a fundamental tool for evaluating the perceptual quality of images by comparing them to a reference standard. This technique is particularly valuable in applications such as image compression, restoration, enhancement, and virtual reality systems [23,24]. Traditional FR-IQA approaches [25,26,27,28,29,30] typically employ handcrafted features and mathematical models to quantify the discrepancies between reference and distorted images. However, these conventional methods often fail to fully align with human visual perception, highlighting the need for advanced techniques that can better mimic human judgment in image quality evaluation.

With the emergence of big data and advances in machine learning, there has been a rapid development of data-driven FR-IQA methods in recent years. These methods utilize machine learning and deep learning models to carry out image quality assessment [31,32,33,34,35], aiming to better capture the complexities of human visual perception. Narwaria and Lin [31] employed features based on singular value decomposition (SVD) and combined them with support vector machines (SVMs). Convolutional neural networks (CNNs) [36,37,38] have proven to be valuable tools in automatically extracting features from image data, making them highly suitable for IQA tasks. Bosse et al. [32] introduced an FR-IQA method known as WaDIQaM, which employs deep neural networks (e.g., VGG architecture) to extract image features and aggregates local patch quality scores via a weighted averaging strategy, addressing the limitations of traditional averaging methods that ignore spatial distortion heterogeneity. As a result, it does not require a priori knowledge about the properties of the human visual system (HVS). Kim and Lee [33] proposed a deep IQA method that learns visually sensitive features for the HVS via a deep CNN. The success of developing deep architectures for image quality assessment, which leverage CNNs and synthetic distortion databases, underscores the importance of capturing the perceptual aspects of image quality through deep neural networks.

The success of developing deep architectures for image quality assessment, which leverage CNNs and synthetic distortion databases, underscores the importance of capturing the perceptual aspects of image quality through deep neural networks [39,40,41,42,43,44]. These models can learn hierarchical features that correspond to different levels of visual information processing in the human visual system. Despite the satisfactory performance of many deep learning-based methods [45,46] on specific datasets, they often struggle to generalize effectively across different types of distortions and image content. This limitation can be attributed to several factors, as follows. First, existing visual quality databases have limited data volumes, which are insufficient for supporting models that can learn the full range of real-life distortion types. Deep learning models may inadvertently overfit the specific types of distortions present in the training data, leading to poor performance on novel distortion types or real-world scenarios not represented in the training set. Second, deep learning-based models tend to be challenging to interpret and often function as “black boxes”. This lack of interpretability makes it difficult to understand the underlying factors contributing to the model’s quality assessment decisions. Third, many deep learning-based IQA models are time-consuming when processing visual information, limiting their applicability in real-time scenarios or large-scale image processing tasks.

**Figure 1:**
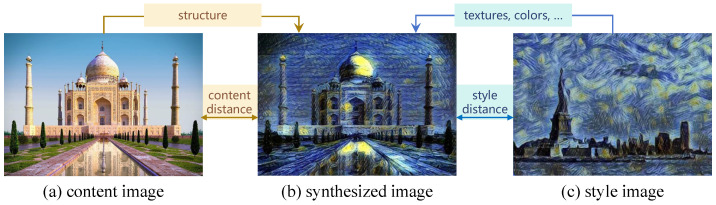
Motivation of the proposed style distance- and content distance-based IQA model (SCIQA).

To address these limitations, IQA methods that can balance the power of deep learning with the interpretability and efficiency of traditional approaches are needed. One promising direction is to incorporate domain knowledge and perceptual principles into the design of deep learning-based IQA models. In this context, the concept of style transfer offers an intriguing perspective on image quality assessment. As shown in Figure 1, style transfer is a technique employed in the realm of computer vision that enables the application of an artistic style from one image to the content of another image, thereby producing synthetic images with innovative artistic effects. The fundamental concept of this technique involves the separation and subsequent recombination of the content and style of two images, resulting in the creation of a novel image.

The content image and the style image undergo feature extraction, with various layers of the network capturing distinct levels of image features. This process of separating content and style information aligns well with the human visual system’s ability to perceive both the structural content and the stylistic elements of an image. Given that an image encompasses structure, color, texture, shape, line, stroke, and other visual components as its primary constituents, the distorted image should mirror the original image in terms of both content and style [47].

Motivated by these considerations, we devised an FR-IQA method, denoted as SCIQA (style- and content-based image quality assessment), from the standpoint of style transfer. This approach aims to leverage the principles of style transfer to create a more comprehensive and perceptually aligned image quality assessment metric. The main contributions of this study are as follows:

- We pioneer an FR-IQA framework inspired by style transfer principles and define a novel deep perceptual distance metric that integrates both content and style features. This dual-component metric comprehensively quantifies distortions across spatial and semantic hierarchies through multilevel feature comparisons.
- We formulate a well-designed convex optimization problem to determine the parameters of the proposed SCIQA model. This optimization approach allows the model to learn from subjective quality assessments while maintaining computational efficiency.
- The proposed SCIQA model exhibits strong interpretability, exceptional prediction accuracy, and low time complexity. These properties make it suitable for a wide range of applications in image processing and computer vision.

The remainder of the paper is organized as follows. Related work is presented in Section 2, while detailed methodological specifics can be found in Section 3. A comprehensive discussion of the experiments is provided in Section 4. Finally, we conclude in Section 5.

## 2. Related Work

The field of FR-IQA has evolved significantly, transitioning from handcrafted feature engineering to sophisticated deep learning architectures. This section reviews the key milestones and methodologies, categorized into traditional, deep learning-based, and emerging approaches, thereby contextualizing the contribution of our proposed method.

### 2.1. Traditional FR-IQA Methods

Early FR-IQA methods were predominantly based on handcrafted features designed to emulate specific aspects of the human visual system (HVS). These approaches can be broadly classified into spatial-domain and transform-domain approaches.

**Spatial-Domain Methods.** These methods operate directly on pixel values to extract perceptual features. The structural similarity index (SSIM) [25] is a seminal work in this category, positing that the HVS is highly adaptable for extracting structural information. It quantifies image degradation by comparing luminance, contrast, and structural components. The influence of SSIM led to numerous extensions, including the multiscale SSIM (MS-SSIM) [26], which incorporates multiscale processing to better mimic the HVS, and the 3-SSIM [27], which assigns greater weight to edge regions. Other notable spatial methods include the feature similarity index (FSIM) [28], which leverages phase congruency, gradient magnitude, and the gradient magnitude similarity deviation (GMSD) [29], an efficient metric based on the standard deviation of a gradient similarity map. Concurrently, the visual saliency index (VSI) [30] demonstrated that incorporating visual saliency maps could further improve alignment with human perception by prioritizing quality assessment in perceptually important regions.

**Transform-Domain Methods.** These approaches analyze images in a transformed space, such as the wavelet or discrete cosine transform (DCT) domains, to model HVS frequency and orientation selectivity. For example, visual information fidelity (VIF) [48] measures the statistical fidelity of an image in the wavelet domain via Gaussian scale mixture models. The visual signal-to-noise ratio (VSNR) [49] is another wavelet-based metric that quantifies distortion visibility thresholds. In the DCT domain, DCT-QM [50] employs an $\ell_{p}$-norm weighted average, offering advantageous mathematical properties such as differentiability. The normalized Laplacian pyramid distance (NLPD) [51] uses a multiscale Laplacian pyramid to systematically analyse visual content across different spatial frequency bands, effectively removing local mean luminance biases.

### 2.2. Deep Learning-Based FR-IQA

The advent of deep learning marked a paradigm shift, enabling models to learn complex, hierarchical features directly from data, often achieving superior performance.

**Feature Learning and End-to-End Models.** Early machine learning approaches combined handcrafted features with regressors such as support vector regression (SVR) [31]. However, modern methods leverage convolutional neural networks (CNNs) for automatic feature learning. WaDIQaM [32] pioneered an end-to-end framework, eliminating the need for manual feature engineering. Similarly, DeepQA [33] uses a CNN to learn visually sensitive features, whereas PieAPP [34] reformulates IQA as a pairwise preference prediction task.

**Transfer Learning and Perceptual Metrics.** To overcome the data scarcity of IQA datasets, transfer learning has become a dominant strategy. Pretrained networks such as VGG-16 and ResNet-50 [39] serve as powerful feature extractors. The Learned Perceptual Image Patch Similarity (LPIPS) [40] metric became a benchmark, demonstrating that the distance between deep features from a pretrained network effectively correlates with human perceptual judgments. Subsequent works built on this idea: DeepSim [52] applied the SSIM concept within the VGG feature space, whereas DeepWSD [41] introduced the Wasserstein distance to compare feature distributions. Comparative studies [42] have further validated the effectiveness of various pretraining strategies.

**Advanced Architectures.** More recent research has focused on developing sophisticated architectures to capture finer perceptual details. DISTS [53] and SWDN [54] improved robustness by explicitly modeling structure and texture similarities and accommodating spatial misalignments. Transformer-based models, such as AHIQ [55], integrate attention mechanisms for superior feature weighting, whereas CVRIQA [56] employs cross-attention to match content-similar patches. TOPIQ [57] introduces a top-down, semantically aware approach, and GDSI [58] models images as graphs to measure topological dissimilarities. Other methods, such as SSHMPQA [59] and ICIQA [60], explore high-order statistical moments and causal inference, respectively, to create more robust and explainable models.

### 2.3. Emerging Trends and Our Position

The landscape of IQA continues to evolve with new paradigms. Fusion-based methods, analogous to “boosting” in machine learning, aim to combine multiple IQA models into a “superevaluator” that leverages their collective strengths [61]. However, their reliance on other methods and high computational overhead have limited their practical adoption.

More recently, the rise of multimodal large language models (MLLMs) has inspired new research directions in IQA [62,63]. These models can provide not only quality scores but also descriptive textual feedback. While powerful, MLLMs are computationally expensive, making them less suitable for applications requiring lightweight deployment and real-time performance, such as in-sensor processing.

### 2.4. Research Gap and Our Contribution

Despite the impressive performance of deep learning models, a critical trade-off has emerged: high accuracy often comes at the cost of interpretability (the “black-box” problem) and computational efficiency. Conversely, traditional methods are efficient and interpretable but often fail to capture the complexity of human perception.

To bridge this gap, our work proposes a hybrid approach that combines the feature representation power of deep networks with clear, perceptually motivated distance metrics. The key innovation of our proposed SCIQA method lies in its unique integration of both the content distance and style distance derived from deep CNN features. While prior work [64] explored comparing feature histograms, which are limited in capturing semantic and structural information, our method offers a more nuanced decomposition. It explicitly formulates IQA via two complementary components:

- **Content Distance.** Structural and semantic discrepancies are measured by computing the direct distance between feature maps.
- **Style distance.** Textural and stylistic differences are captured by calculating the distance between Gram matrices, which represent feature correlations.

This dual-component framework allows SCIQA to perform a comprehensive evaluation that considers both high-level semantic structure and low-level textural details, leading to a more robust and perceptually aligned assessment that remains computationally efficient and interpretable.

## 3. Methodology

### 3.1. Framework

**Figure 2:**
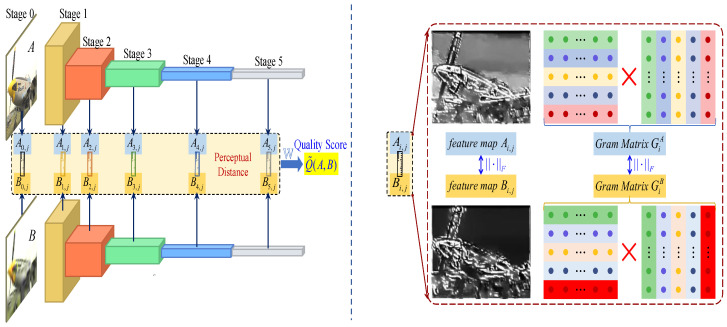
The framework of the proposed FR-IQA model uses the VGG-16 network to generate deep feature maps at stages 0–5. By calculating the content distance and style distance, the model predicts the quality of the distorted image in comparison to the reference image.

As illustrated in Figure 2, we propose a novel style-transfer-inspired full-reference image quality assessment (FR-IQA) framework. The proposed architecture comprises three principal components:

First, we employ a pretrained VGG-16 network for hierarchical feature extraction from both reference and distorted images (left panel in Figure 2). This deep feature extraction mechanism captures multiscale visual characteristics, spanning low-level texture details to high-level semantic information [8,65]. Second, we introduce a novel perceptual dissimilarity metric that integrates both structural content preservation and style consistency measures (right panel in Figure 2). This dual-component metric enables a comprehensive quantification of visual degradation through structural content deviations measured via deep feature correlations and style discrepancies quantified through Gram matrix statistics. Third, we formulate a convex optimization framework for parameter estimation in the proposed SCIQA model. This regularized least-squares formulation ensures stable numerical solutions while maintaining computational efficiency.

Our method is characterized by its simplicity, explainability, and efficiency. By leveraging pretrained networks and convex optimization, we eliminate the need for extensive training, making our approach more practical and easier to implement than end-to-end deep learning methods.

### 3.2. Perceptual Dissimilarity Metric

#### 3.2.1. Hierarchical Feature Representation

Let $A={⨁}_{c=1}^{3}A_{0,c}$ and $B={⨁}_{c=1}^{3}B_{0,c}$ denote reference and distorted images, respectively, where ⊕ represents channelwise concatenation. The subscripts represent the three color channels of red, green, and blue (RGB). Through VGG-16 forward propagation, we extract multistage feature representations:

(1) $$F_{A}=\left\{A_{i,j}\in R^{H_{i}\times W_{i}}|i\in \left\{1,\ldots ,5\right\},j\in \left\{1,\ldots ,L_{i}\right\}\right\}$$

(2) $$F_{B}=\left\{B_{i,j}\in R^{H_{i}\times W_{i}}|i\in \left\{1,\ldots ,5\right\},j\in \left\{1,\ldots ,L_{i}\right\}\right\}$$

where $A_{i,j}$ corresponds to the *j*-th channel of the feature map obtained at the *i*-th stage and where $B_{i,j}$ is similar to $A_{i,j}$. $L_{i}$ denotes the channel dimensions at the *i*-th stage with $\left(L_{1}\ldots ,L_{5}\right)=\left(64,128,256,512,512\right)$. The spatial dimensions $\left(H_{i},W_{i}\right)$ are progressively reduced through max pooling operations.

#### 3.2.2. Structural Content Preservation

Content loss functions are crucial in training deep neural networks for various image processing tasks, including style transfer, image superresolution, and generative adversarial networks (GANs). These functions quantify the disparity between the content of two images by leveraging feature representations from a pretrained convolutional neural network (CNN).

In computer vision, image content primarily consists of an image structure. As we progress through deeper stages of the network, the representations tend to offer fewer details but more pronounced shape and structural information. To this end, we employ the Frobenius matrix norm to define the content distance between *A* and *B* as follows:

(3) $$d_{\mathrm{content}}\left(A,B\right)=\sum_{i=1}^{5}\sum_{j=1}^{L_{i}}\alpha_{i,j}{∥A_{i,j}-B_{i,j}∥}_{F}$$

where $\alpha_{i,j}$ are learnable parameters controlling hierarchical importance. Deeper layers (*i* increasing) emphasize semantic content, whereas shallower layers capture low-level details.

#### 3.2.3. Style Consistency Measurement

Image texture refers to the repetitive patterns, intricate details, or structures present in an image, encompassing variations in geometric shapes, colors, brightness, and other attributes. Image style transfer involves adjusting various aspects of an image, such as colors, lines, and strokes, to achieve a particular artistic effect.

The image style and image texture can be regarded as expressions of the same entity in different domains. Image texture is more relevant to academic fields such as computer vision, whereas image style is more pertinent to the domain of artistic painting. Therefore, we can measure the texture difference between two images via a style distance metric.

Inspired by the findings of Gatys et al. [66] and Johnson et al. [67], we define the style distance between *A* and *B* as follows:

(4) $$d_{\mathrm{style}}\left(A,B\right)=\sum_{i=1}^{5}\beta_{i}{∥G_{i}^{A}-G_{i}^{B}∥}_{F}$$

where $\beta_{i}$ are style importance weights. The Gram matrix $G_{i}^{I}\in R^{L_{i}\times L_{i}}$ for image *I* at stage *i* is computed as

(5) $$G_{i}^{I}=\frac{1}{H_{i}W_{i}}\Psi_{i}^{I}{\left(\Psi_{i}^{I}\right)}^{⊤},\;\Psi_{i}^{I}=\left[\mathrm{vec}\left(I_{i,1}\right)\cdots \mathrm{vec}\left(I_{i,L_{i}}\right)\right]$$

where vec(·) denotes spatial vectorization. This formulation captures channelwise feature correlations that represent texture information.

### 3.3. SCIQA Model Formulation

We construct the proposed SCIQA model by combining content distance and style distance, incorporating a bias term as follows:

(6) $$\hat{Q}\left(A,B\right)=\underset{\mathrm{Content}\;\mathrm{Term}}{\underset{︸}{\sum_{i,j}\alpha_{i,j}{∥A_{i,j}-B_{i,j}∥}_{F}}}+\underset{\mathrm{Style}\;\mathrm{Term}}{\underset{︸}{\sum_{i}\beta_{i}{∥G_{i}^{A}-G_{i}^{B}∥}_{F}}}+b$$

where $\hat{Q}$ denotes the quality score predicted by the model. The content term measures feature discrepancies across spatial positions (i,j) and channel dimensions through Frobenius norms. The style term quantifies the Gram matrix differences $G_{i}$ at different CNN layers. The bias term *b* provides baseline quality calibration. Parameters $\alpha_{i,j}$ and $\beta_{i}$ weight the relative importance of different perceptual distances.

Assume that an FR-IQA database comprises *M* pairs of reference and distorted images, designated as $\left\{\left(A^{1},B^{1}\right),\ldots ,\left(A^{k},B^{k}\right),\ldots ,\left(A^{M},B^{M}\right)\right\}$, with $Q^{k}$ denoting the true perceptual quality of $B^{k}$. We formulate a perceptual optimization problem as follows:

(7) $$\min_{w}{∥Fw-q∥}_{2}^{2}+\lambda {∥w∥}_{2}^{2}$$

where $w={\left[\alpha_{1,1},\ldots ,\alpha_{5,512},\beta_{1},\ldots ,\beta_{5},b\right]}^{⊤}$ contains 1482 parameters, and $q=[Q^{1},$$\ldots ,Q^{M}{]}^{⊤}$. The construction of $F\in R^{M\times 1482}$ is as follows:

(8) $$F\left[k,:\right]=[\{∥A_{i,j}^{k}-B_{i,j}^{k}{∥}_{F}\},\{∥G_{i}^{A^{k}}-G_{i}^{B^{k}}{∥}_{F}\},1]$$

The optimization aims to minimize both prediction errors (${∥\mathrm{Fw}-q∥}_{2}^{2}$) and parameter magnitudes (${\lambda ∥w∥}_{2}^{2}$), where F encodes pairwise feature differences for all *M* image pairs. Each row of F contains the following: content differences $∥A_{i,j}^{k}-B_{i,j}^{k}{∥}_{F}$ for all feature maps, style differences $∥G_{i}^{A^{k}}-G_{i}^{B^{k}}{∥}_{F}$ across CNN layers, and a unity placeholder for the bias parameter.

The global optimum parameter vector can be obtained via the following equation:

(9) $$\hat{w}={\left(F^{⊤}F+\lambda I\right)}^{-1}F^{⊤}q$$

where I is the 1482×1482 identity matrix. The closed-form solution derives from ridge regression theory, where ${\left(F^{⊤}F+\lambda I\right)}^{-1}$ regularizes the feature covariance matrix to ensure numerical stability. The identity matrix I shares dimensionality with the parameter space (1482 × 1482), and λ controls the regularization strength. Thus, we can efficiently compute the optimal parameters for the proposed SCIQA model.

From a mathematical standpoint, Equation (7) constitutes a convex optimization problem that inherently guarantees the existence of a global optimal solution, regardless of the dataset size *M*. However, practical implementation and solution quality depend critically on the structural properties of the matrix F. The optimization framework involves 1482 parameters, with the dimensionality of the matrix F being M×1482. To ensure that matrix F contains sufficient information for reliable parameter estimation, its row space must satisfy the rank condition: rank(F)=1482.

This rank requirement implies that the number of data points *M* should theoretically satisfy M≥1482. When M<1482, the matrix becomes rank deficient (rank(F)=1482), leading to an underdetermined system. In contrast, when M≥1482, the matrix F can achieve maximum rank, ensuring that the optimization problem is well-posed to fully exploit the parameter space. This dimensional analysis aligns with the fundamental principle in linear algebra that the rank of a matrix cannot exceed its smaller dimension. The 1482-parameter structure therefore imposes a theoretical lower bound on the required sample size for faithful model reconstruction.

Following the acquisition of W, the proposed method can be implemented as described in Algorithm 1. This algorithm involves simple matrix and vector operations, resulting in low computational complexity. Moreover, it can be further accelerated through parallel processing techniques, leveraging both algorithmic and hardware optimizations.
**Algorithm 1** The proposed SCIQA Model 1:input: A pair of reference and distorted images (A,B) 2:output: The quality of distorted image *B* in comparison to the reference image *A* 3:**for** i=0 to 5 **do** 4:   $G_{i}^{A}=\left[\;\right]$ 5:   $G_{i}^{B}=\left[\;\right]$ 6:   **for** j=1 to $N_{i}$ **do** 7:     $d_{\mathrm{content}}^{i,j}={∥A_{i,j}-B_{i,j}∥}_{F}$ 8:     $G_{i}^{A}=\left[G_{i}^{A};flatten\left(A_{i,j}\right)\right]$ 9:     $G_{i}^{B}=\left[G_{i}^{B};flatten\left(B_{i,j}\right)\right]$10:   **end for**11:   $G_{i}^{A}=G_{i}^{A}\cdot {\left(G_{i}^{A}\right)}^{T}$12:   $G_{i}^{B}=G_{i}^{B}\cdot {\left(G_{i}^{B}\right)}^{T}$13:   $d_{\mathrm{style}}^{i}={∥G_{i}^{A}-G_{i}^{B}∥}_{F}$14:**end for**15:$f=[\left\{d_{\mathrm{content}}^{i,j}\right\},\left\{d_{\mathrm{style}}^{i}\right\},1]$16:$\hat{Q}=f^{⊤}w$17:**return** $\hat{Q}$

In summary, the proposed SCIQA model offers a novel approach to FR-IQA by combining content and style distances derived from deep feature representations. The SCIQA model exhibits architectural simplicity, theoretical explainability, and computational efficiency. It leverages pretrained features without complex network designs. Explicit perceptual distance formulation enables human interpretation. The convex optimization formulation ensures global optimality and eliminates iterative training requirements, making it a practical solution for assessing image quality in various applications.

## 4. Experimental Results

This section presents a comprehensive evaluation of the proposed SCIQA model, including detailed implementation specifications, performance comparisons with state-of-the-art methods, an analysis of the model’s sensitivity to hyperparameters, and a subjective objective consistency experiment.

### 4.1. Implementation Details

To ensure a rigorous evaluation of the SCIQA model, we utilized five widely recognized subjective quality assessment databases: LIVE [68], CSIQ [69], TID2013 [70], KADID-10k [71], and PIPAL [72]. These datasets were chosen for their diversity in image content, distortion types, and subjective rating methodologies, providing a robust foundation for assessing the model’s performance across various scenarios.

Developed by the University of Texas at Austin, the LIVE dataset is one of the most widely used benchmarks for image quality assessment. It consists of 29 reference images and 779 distorted images, typically with a resolution of 768×512 pixels. The dataset includes five types of computer-generated distortions: JPEG2000 compression (175 images), JPEG compression (169 images), white noise (145 images), Gaussian blur (145 images), and fast fading (145 images). Subjective evaluations are based on differential mean opinion scores (DMOSs) ranging from 0–100, which are derived from approximately 25,000 ratings provided by 161 observers. Created by Oklahoma State University, the CSIQ dataset focuses on categorical subjective quality assessment. It contains 30 reference images and 866 distorted images, all standardized to 512×512 pixels. The six distortion types include JPEG compression, JPEG2000 compression, global contrast reduction, additive Gaussian pink noise, additive Gaussian white noise, and Gaussian blur. The DMOS scores range from 0 to 1 and are calculated from approximately 5000 subjective ratings by 25 observers. As an extension of TID2008, the TID2013 dataset aims to cover a broader range of distortion types. It comprises 25 reference images and 3000 distorted images at 512×384 resolution. The dataset features 24 distortion categories, including additive noise, quantization noise, and JPEG transmission errors. Subjective evaluations use mean opinion scores (MOSs) obtained through large-scale experiments. As one of the largest artificially distorted datasets, KADID-10k includes 81 reference images and 10,125 distorted images, with each reference image generating 125 distorted versions. It covers 25 distortion types, each with 5 severity levels. The MOS-based evaluation makes it suitable for large-scale algorithm validation. PIPAL is one of the most comprehensive benchmarks. It contains 250 reference images and 25,850 distorted images, spanning 40 distortion types. The key features include traditional distortions and GAN-generated artifacts. The dataset employs an Elo rating system for dynamic subjective scoring, enhancing reliability through pairwise comparisons. The key features are summarized in Table 1.

In our implementation, we maintain the convolutional kernels within the deep VGG-16 network as fixed, leveraging the pretrained weights on ImageNet. The choice of using a pretrained VGG-16 network stems from its well-established ability to capture rich and discriminative visual features across a wide range of image domains. The VGG-16 network, pretrained on the ImageNet dataset, has been trained on millions of diverse natural images, enabling it to learn hierarchical features that generalize well to unseen domains. This is particularly advantageous for image quality assessment (IQA), where the training datasets (e.g., KADID-10k, PIPAL) are relatively small compared with large-scale datasets such as ImageNet. This decision was made to capitalize on the rich feature representations learned from a diverse set of natural images, which has been shown to transfer well to image quality assessment tasks [40,53]. The regularization factor λ in Equation (7) was set to 75 after a series of experiments to determine the optimal value, as discussed in detail in Section 4.3. The KADID-10k dataset was intentionally selected as our primary training set because of its well-balanced characteristics as a moderate-scale database containing approximately 10,125 annotated images.

To evaluate the performance of the SCIQA method rigorously, three standard correlation coefficients are employed to compare the predicted and ground-truth quality scores: the Pearson linear correlation coefficient (PLCC), the Spearman rank-order correlation coefficient (SROCC), and the Kendall rank-order correlation coefficient (KROCC). In cases where the predicted quality scores and subjective scores exhibit disparate value domains, we employ the nonlinear five-parameter logistic (5PL) function [73] to fit the ground-truth scores before computing the PLCC, as follows:

(10) $$\tilde{Q}=f\left(Q;p\right)=f\left(Q;a,b,c,d,g\right)=d+\frac{\left(a-d\right)}{{\left(1+{\left(\frac{Q}{c}\right)}^{b}\right)}^{g}},$$

where p=[a,b,c,d,e] denotes the parameter vector of the 5PL to be estimated.

### 4.2. Performance Comparison

In this subsection, we conducted a performance comparison of the proposed SCIQA model against 18 commonly utilized or state-of-the-art methods, including PSNR, SSIM [25], MS-SSIM [26], VIF [48], FSIM [28], VSI [30], GMSD [29], NLPD [51], WaDIQaM [32], PieAPP [34], LPIPS [40], SWDN [54], DeepWSD [41], DISTS [53], CVRIQA [56], TOPIQ [57], SSHMPQA [59], and ICIQA [60]. Notably, these methods employ diverse technical approaches, ranging from traditional feature engineering (GMSD, VSI) to deep learning frameworks (LPIPS, DISTS) and recent hybrid architectures. In addition, both the LPIPS and DISTS methods employ a pretrained VGG-16 network for visual feature extraction.

**Figure 3:**
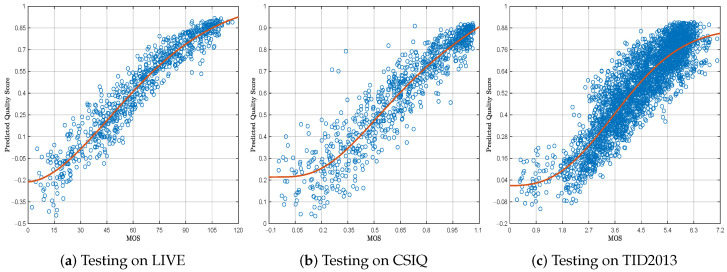
Scatter plots and 5PL fitting curves between the predicted quality scores and the subjective quality scores on the testing datasets. The blue circle represents the scatter plots, and the red circle represents the 5PL fitting curve.

The comparative results on three small-scale datasets (LIVE, CSIQ, and TID2013) are presented in Table 2, where the three best values in each column are bolded in red, green and blue. The experimental results show that the proposed SCIQA model is among the top three for a total of nine criteria across the three datasets, illustrating its robust generalization ability. As depicted in Figure 3, a strong correlation emerges between the quality scores predicted through the SCIQA methodology and the ground-truth perceptual quality assessments.

For comprehensive performance evaluation, we also adopted validation on a large-scale PIPAL dataset to examine scalability generalization capabilities and robustness under high-volume real-world conditions. As shown in Table 3, our method achieves state-of-the-art performance compared with both traditional and deep learning-based approaches, which proves its generalizability and robustness. The SCIQA framework has the highest SROCC score of 0.702 and a competitive PLCC of 0.705. This performance across both metrics also indicates superior consistency with human subjective judgments.

VSI and GMSD achieve high scores on small-scale datasets such as LIVE and CSIQ (e.g., GMSD’s SROCC = 0.939 on CSIQ) because their handcrafted designs target specific distortion types. However, their performance degrades significantly on larger/more diverse datasets (e.g., GMSD’s SROCC = 0.804 on TID2013 and 0.569 on PIPAL), revealing poor generalizability.

Traditional methods such as VSI/GMSD rely on shallow handcrafted features (e.g., gradient/contrast measures) that cannot capture high-level semantic distortions or complex style-texture interactions. This limitation becomes pronounced in modern applications involving more complex distortions. In contrast, SCIQA achieves state-of-the-art performance on the large-scale PIPAL dataset (SROCC = 0.702 vs. GMSD’s 0.569 and VSI’s 0.526) while maintaining balanced accuracy across all benchmarks.

### 4.3. Parameter Sensitivity

**Figure 4:**
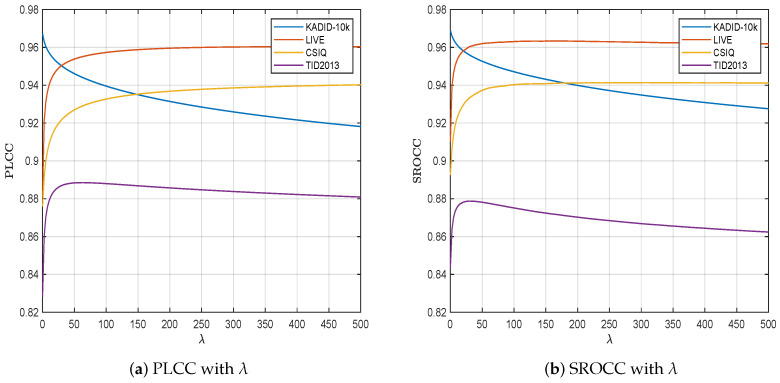
IQA performance variations in the PLCC and SROCC with λ.

The SCIQA model has only one hyperparameter λ, which controls the strength of regularization in the convex optimization problem. To determine the optimal value of λ, we conducted a grid search over the range λ∈[0,500] on the training set of the KADID-10k dataset and present the variations in terms of the PLCC and SROCC with respect to λ in Figure 4.

As the optimization problem in (7) transitions from having no regularization term to incorporating one, the model’s performance significantly improves on the testing set. Notably, when the regularization factor exceeds 25, the model’s performance stabilizes, indicating a reduced sensitivity to this parameter. To achieve an optimal balance in the SCIQA model’s performance across all datasets, it is advisable to set λ within the range of [50, 100]. In this work, a value of λ=75 is chosen to provide an optimal balance between minimizing prediction errors and preventing overfitting.

### 4.4. Ablation Study

#### 4.4.1. VGG-16 Network

To validate the effectiveness of using a pretrained VGG-16 network, we conducted additional experiments by training a VGG-16 network from scratch on the KADID-10k dataset and testing it on the LIVE dataset. The results, as shown in Table 4, demonstrate that the pretrained network consistently outperforms the self-trained network across all the metrics.

When trained from scratch on IQA-specific datasets, the network may struggle to learn robust feature representations because of the limited data size and diversity. This can lead to overfitting and reduced generalization performance across different distortion types and image contents. Thus, the pretrained network achieves significantly higher PLCC, SROCC, and KROCC scores than the self-trained network does. This finding validates our hypothesis that the pretrained network’s ability to generalize across diverse visual domains is critical for achieving robust performance in IQA tasks.

#### 4.4.2. Perceptual Distances

To rigorously evaluate the contributions of image content distance ($d_{content}$) and perceptual style distance ($d_{style}$) to the performance of our proposed image quality assessment model, SCIQA, we conducted a comprehensive ablation study across three benchmark datasets: LIVE, CSIQ, and TID2013. The results are summarized in Table 5, where we compare the original SCIQA model against two ablated variants: a model without the content distance component and a model without the style distance component.

As we can see, removing content distance causes severe performance degradation across all datasets, confirming that content fidelity is fundamental to human quality perception. Ablating style distance leads to moderate but consistent decreases. The smaller performance gap implies that style features refine predictions but are less decisive than content features. This aligns with the cognitive principle that humans prioritize semantic content integrity over stylistic details when evaluating quality.

### 4.5. Time and Complexity Analysis

We conducted a comprehensive computational complexity evaluation to further assess the efficiency of our proposed SCIQA method. The computational complexity is analyzed in terms of both time and space requirements, and the results are compared with those of state-of-the-art methods.

The SCIQA model consists of three main components: feature extraction, style distance computation, and content distance computation. The feature extraction stage leverages a pretrained VGG-16 network, which has a time complexity of O(N·H·W), where *N* is the number of channels, and *H* and *W* are the spatial dimensions of the feature maps. The style distance computation involves calculating Gram matrices for each feature map, with a time complexity of $O(L^{2}\cdot H\cdot W)$, where *L* is the number of channels. The content distance computation calculates the Frobenius norm between corresponding feature maps, with a time complexity of O(L·H·W). In terms of space complexity, the SCIQA model requires storing the feature maps and Gram matrices, resulting in a space complexity of O(L·H·W) for feature maps and $O(L^{2}\cdot H\cdot W)$ for Gram matrices.

To provide a fair comparison, we evaluated the computational efficiency of our method against several state-of-the-art IQA methods, including SSHMPQA [59], DISTS [53], and LPIPS [40]. The results are summarized in Table 6.

As shown in Table 6, our SCIQA method achieves superior inference speed compared with existing methods while maintaining comparable computational complexity. The significant improvement in speed, combined with competitive accuracy, makes SCIQA a practical solution for real-time applications where rapid processing is critical.

### 4.6. Subjective Consistency Experiments

**Figure 5:**
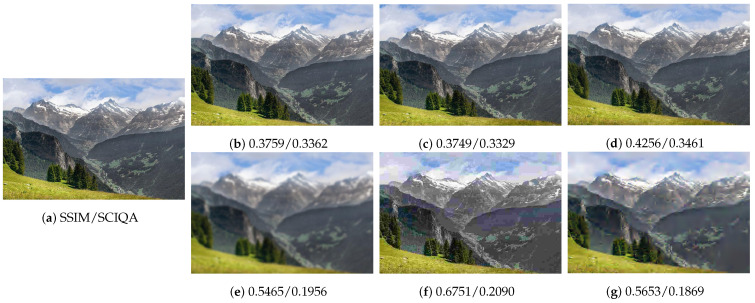
Comparative analysis of quality assessment robustness under spatial perturbations. (**a**) reference image; (**b**) 1.05× magnification; (**c**) 3-degree rotation; (**d**) cloud displacement; (**e**) Gaussian blur; (**f**) JPEG compression; and (**g**) JPEG2000 compression. While the conventional SSIM erroneously predicts higher-quality scores for compression artifacts (**e**–**g**), the proposed SCIQA framework demonstrates superior alignment with human perception by correctly identifying the perceptual superiority of geometrically transformed images (**b**–**d**) over compression-degraded counterparts.

A subjective consistency experiment was conducted to evaluate the robustness of the image quality assessment methods, as illustrated in Figure 5. The test set comprises seven distinct image conditions. Both the SSIM [25] and our proposed SCIQA quality scores are displayed beneath each processed image, with visual artifacts becoming more apparent upon image magnification.

The experimental results reveal a critical limitation in SSIM’s ability to handle spatial transformations, where it assigns disproportionately low scores (0.3362–0.3461) to perceptually similar variants (Figure 5b–d) while failing to adequately penalize compression artifacts (0.1869–0.2090). In contrast, the proposed SCIQA method demonstrates both improved sensitivity to compression artifacts and enhanced robustness to geometric transformations, better reflecting human visual preferences.

### 4.7. Interpretability Experiments

**Figure 6:**
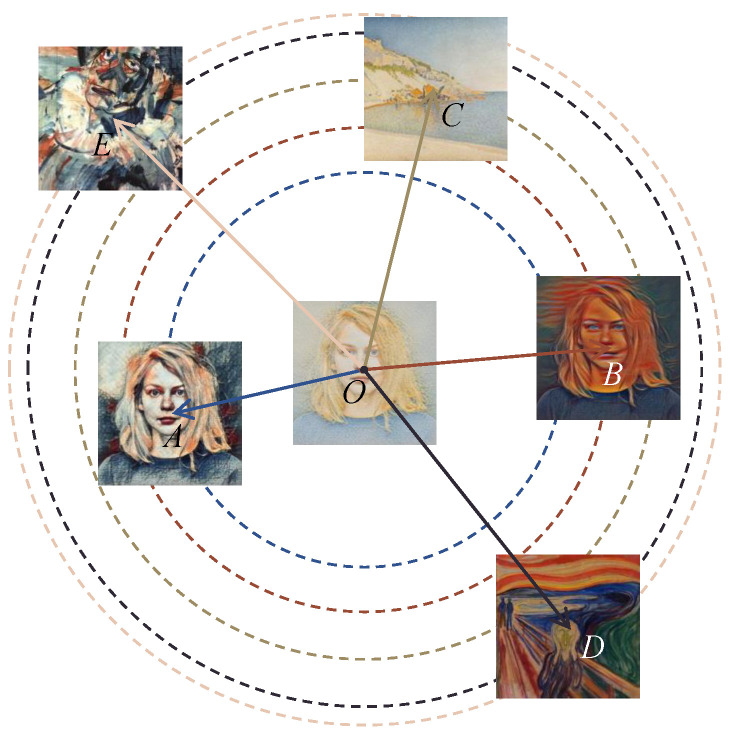
Illustration of the interpretability of the SCIQA method. Let d(X,Y)=1−SCIQA(X,Y) denote the distortion distance between *X* and *Y*. On the basis of this definition, we calculate the following distortion distances from *O*: d(O,A)=0.4766, d(O,B)=0.4898, d(O,C)=0.6970, d(O,D)=0.8398, and d(O,E)=0.8614.

As illustrated in Figure 6, we select three sets of images, each consisting of a style image and its corresponding portrait. Specifically, C and O form one pair, B and D form another, and E and A form the third pair. We used the portrait O as the reference image, with the others considered distorted images. By applying the proposed SCIQA method to calculate the perceptual distances, we observe that the images closest to O are portraits A and B, followed by their corresponding style image C. The images farthest from O are style images D and E, which have no semantic relation to O.

This observation indicates that the proposed SCIQA method effectively perceives the content of objects in the images. Portraits O, A, and B depict the same person and thus share identical semantic content, which is the most crucial information in an image. Consequently, A and B are closest to O in terms of perceptual distance. Additionally, the similarity in style between C and O results in a relatively short distance between them, demonstrating that our method also accurately captures image textures.

The results of this experiment underscore the high interpretability of the proposed SCIQA method. It not only effectively discerns the semantic content of images but also recognizes stylistic similarities, making it a robust tool for image quality assessment.

### 4.8. Discussion

#### 4.8.1. Comparison Between SSHMPQA and SCIQA

To clarify the differences between SSHMPQA [59] and SCIQA, we present a concise comparative analysis, highlighting the advantages of SCIQA. In terms of computational efficiency, SCIQA significantly outperforms SSHMPQA. While SSHMPQA requires 728 min for feature extraction on the KADID-10k dataset, SCIQA achieves this in just 10 min. Furthermore, SCIQA completes training in 0.186 s and has an inference speed of 23.6 frames per second (fps), which is 30 times faster than that of SSHMPQA, with comparable accuracy. Methodologically, SSHMPQA employs high-order statistical moments for texture analysis, which, while effective, involves extensive computations. SCIQA adopts a style-transfer-inspired approach, measuring structural discrepancies through content distance and texture differences via style distance via Gram matrices. This approach avoids explicit structure–texture decomposition and iterative optimization, reducing complexity. Although SSHMPQA shows marginally higher accuracy in certain metrics, SCIQA offers a superior balance between accuracy, speed, and generalizability, making it more practical for real-world applications.

#### 4.8.2. Performance Comparison and Analysis

The experimental results demonstrate that the proposed SCIQA model achieves state-of-the-art performance across multiple benchmark datasets, outperforming both traditional and deep learning-based image quality assessment (IQA) methods. Traditional IQA methods often fail to fully align with human visual perception, particularly when dealing with complex distortions or diverse image content. For example, GMSD, which focuses on gradient magnitude similarity, performs well on small-scale datasets such as LIVE and CSIQ (SROCC = 0.939 on CSIQ) but struggles to generalize to larger, more diverse datasets such as PIPAL (SROCC = 0.569). This limitation highlights the challenge of designing handcrafted features that can capture the full range of perceptual aspects of image quality. Deep learning-based methods suffer from challenges such as overfitting to specific distortion types, a lack of interpretability, and high computational complexity. For example, LPIPS, which measures perceptual differences through deep feature representations, achieves strong performance on small-scale datasets (PLCC = 0.934 on LIVE) but struggles to maintain consistency on large-scale datasets such as PIPAL (PLCC = 0.633). This indicates the need for methods that balance the power of deep learning with interpretability and efficiency.

The proposed SCIQA model addresses these limitations by combining the strengths of deep learning with perceptually inspired principles. By leveraging the VGG-16 network for hierarchical feature extraction and incorporating both content and style distances, SCIQA achieves a comprehensive and interpretable measure of image quality. The integration of content distance, which captures structural and semantic information, and style distance, which quantifies texture and visual characteristics, enables SCIQA to better mimic human visual perception. This dual-component metric not only improves prediction accuracy but also enhances interpretability, as demonstrated in the ablation study and subjective consistency experiments. The superior performance of SCIQA across multiple datasets, including small-scale benchmarks (e.g., PLCC = 0.956 on LIVE) and large-scale datasets (e.g., SROCC = 0.702 on PIPAL), highlights its robust generalization ability.

#### 4.8.3. Limitations and Prospects

While the proposed SCIQA method demonstrates promising performance in image quality assessment, it is important to acknowledge its limitations and consider future research directions.

***Limitations.*** The SCIQA method relies on a pretrained VGG-16 network trained on general-purpose datasets such as ImageNet. While this approach leverages transfer learning to capture robust visual features, it may limit the model’s ability to adapt to specific domains or datasets with unique characteristics. The SCIQA method has not yet been validated in specific application domains, such as medical imaging or autonomous systems, where image quality assessment may require domain-specific considerations. Although SCIQA incorporates interpretable components such as content and style distances, the deep feature extraction process may still exhibit some degree of “black-box” behavior, making it challenging to fully explain all aspects of the model’s decisions.

***Prospects.*** The SCIQA framework could be extended to video quality assessment by incorporating temporal feature analysis. This would involve developing spatiotemporal feature representations to capture motion-related distortions and temporal consistency. To address computational complexity, future work could focus on optimizing the SCIQA model for real-time applications. This could involve lightweight network architectures, hardware acceleration, or model compression techniques. The SCIQA method can be adapted to specific domains, such as medical imaging or satellite imagery, by fine-tuning the pretrained VGG-16 network on domain-specific datasets. This enables the model to better capture domain-specific features and distortions. Future research could focus on improving the interpretability of the SCIQA method by developing visualization techniques for deep features and providing more detailed explanations of the content and style distance calculations.

## 5. Conclusions

In this paper, we present SCIQA, a novel FR-IQA model developed from the perspective of style transfer. Our approach uniquely integrates both image content and style, addressing the gap between traditional IQA methods and modern deep learning techniques. The SCIQA model is both knowledge-driven and data-driven and is established through rigorous mathematical analytic methods. Experimental results across multiple benchmark datasets demonstrate that SCIQA achieves superior prediction accuracy, strong interpretability, and low computational complexity compared with state-of-the-art FR-IQA methods. Specifically, SCIQA achieves Pearson linear correlation coefficients (PLCCs) of 0.956, 0.941, and 0.895 on the LIVE, CSIQ, and TID2013 datasets, respectively, outperforming traditional methods such as SSIM (PLCC: 0.847, 0.852, 0.665) and deep learning-based methods such as DISTS (PLCC: 0.924, 0.919, 0.855). The proposed method also demonstrates robust generalizability on the large-scale PIPAL dataset, achieving an SROCC of 0.702. These results underscore the superior performance and reliability of SCIQA in capturing human visual perception across diverse distortion types and image contents.

The proposed method has the potential to be extended to no-reference IQA and video quality assessment tasks in future work, further broadening its applicability in sensor-related domains. To facilitate reproducibility and encourage further research in this area, the source code for the proposed model is publicly available at https://github.com/Math-Computer/SCIQA (accessed on 10 August 2025).

## Figures and Tables

**Table 1:** Summary of the key features of the five datasets.

Feature	LIVE	CSIQ	TID2013	KADID-10k	PIPAL
Reference Images	29	30	25	81	250
Distorted Images	779	866	3000	10,125	25,850
Distortion Types	5	6	24	25	40
Evaluation Method	DMOS	DMOS	MOS	MOS	Elo
Primary Focus	Traditional	Traditional	Multitype	Multilevel	Multitype

**Table 2:** Evaluation results on three small-scale datasets: LIVE, CSIQ, and TID2013. The first-, second- and third-best results in each column are recorded as bold red, green, and blue, respectively.

Method	Dataset	LIVE			CSIQ			TID2013		
Category	Criterion	PLCC	ROCC	KROCC	PLCC	SROCC	KROCC	PLCC	SROCC	KROCC
Traditional Methods	PSNR	0.781	0.801	0.677	0.792	0.807	0.603	0.664	0.687	0.496
	SSIM	0.847	0.851	0.789	0.852	0.865	0.680	0.665	0.627	0.545
	MS-SSIM	0.886	0.903	0.805	0.875	0.879	0.730	0.830	0.786	0.605
	VIF	**0.949**	0.953	**0.817**	0.899	0.879	0.743	0.771	0.677	0.518
	FSIM	0.910	0.920	0.806	0.875	0.884	0.769	0.877	0.851	0.667
	VSI	0.877	0.899	0.806	0.902	0.915	0.786	**0.898**	**0.895**	**0.718**
	GMSD	0.909	0.910	0.787	**0.938**	**0.939**	**0.804**	0.855	0.804	0.634
	NLPD	0.882	0.889	0.758	0.913	0.926	0.749	0.839	0.800	0.625
Deep Learning- Based Methods	WaDIQaM	0.940	0.947	0.791	0.901	0.909	0.732	0.834	0.831	0.631
	PieAPP	0.866	0.865	0.740	0.864	0.883	0.712	0.859	0.876	0.683
	LPIPS	0.934	0.932	0.765	0.896	0.876	0.689	0.749	0.670	0.497
	DeepWSD	0.904	0.925	0.813	0.941	0.950	0.812	0.894	0.874	0.783
	DISTS	0.924	0.925	0.807	0.919	0.920	0.746	0.855	0.830	0.639
	CVRIQA	0.944	**0.954**	0.811	0.903	0.869	0.689	0.734	0.732	0.546
	TOPIQ	0.882	0.887	0.775	0.894	0.893	0.789	0.854	0.820	0.664
	SSHMPQA	**0.959**	**0.963**	**0.828**	**0.945**	**0.945**	**0.795**	**0.897**	**0.879**	**0.694**
2cSCIQA (ours)		**0.956**	**0.964**	**0.828**	**0.941**	**0.940**	**0.798**	**0.895**	**0.877**	**0.690**

**Table 3:** Evaluation results on a large-scale dataset PIPAL. The first-, second-, and third-best results in each column are recorded as bold red, green, and blue, respectively.

Category	Method	PLCC	SROCC
Traditional Methods	PSNR	0.398	0.392
	SSIM	0.489	0.486
	MS-SSIM	0.571	0.545
	VIF	0.572	0.545
	MAD	0.614	0.591
	FSIM	0.597	0.573
	VSI	0.548	0.526
	GMSD	0.614	0.569
	NLPD	0.489	0.464
Deep Learning- Based Methods	PieAPP	0.597	0.607
	LPIPS	0.633	0.595
	SWDN	0.634	0.624
	DISTS	0.687	0.655
	SSHMPQA	**0.709**	**0.692**
	ICIQA	**0.694**	**0.656**
SCIQA (ours)		**0.705**	**0.702**

**Table 4:** Comparison of IOA performance on the LIVE dataset using a pretrained VGG-16 network versus a self-trained VGG-16 network on the KADID-10k dataset. The best scores are marked in blod.

Criterion	PLCC	SROCC	KROCC
Pretrained VGG-16	**0.956**	**0.964**	**0.828**
Self-trained VGG-16	0.795	0.789	0.624

**Table 5:** Ablation study results. In each column, the best value is marked in bold.

Dataset	LIVE			CSIQ			TID2013		
Criterion	PLCC	SROCC	KROCC	PLCC	SROCC	KROCC	PLCC	SROCC	KROCC
original SCIQA	**0.956**	**0.964**	**0.828**	**0.941**	**0.940**	**0.798**	**0.895**	**0.877**	**0.690**
W/O $d_{content}$	0.827	0.816	0.691	0.811	0.803	0.655	0.793	0.788	0.546
W/O $d_{style}$	0.917	0.928	0.779	0.924	0.916	0.776	0.836	0.825	0.624

**Table 6:** Computational efficiency comparison of different IQA methods. The best performance is marked in bold.

Method	Inference Speed (FPS)
SSHMPQA	0.76
DISTS	12.3
LPIPS	18.5
**SCIQA (ours)**	**23.6**

## Data Availability

The original contributions presented in the study are included in the article, and further inquiries can be directed to the corresponding authors.

## References

[1] Lee C., Kim D., Kim D. (2025). Quality Assessment of High-Speed Motion Blur Images for Mobile Automated Tunnel Inspection. Sensors.

[2] Chen Z., Du J., Li J., Lv H. (2025). MDFN: Enhancing Power Grid Image Quality Assessment via Multi-Dimension Distortion Feature. Sensors.

[3] Rotter P., Knapik D., Klemiato M., Rosół M., Putynkowski G. (2025). Compensation of Speckle Noise in 2D Images from Triangulation Laser Profile Sensors Using Local Column Median Vectors with an Application in a Quality Control System. Sensors.

[4] Wu H., Zeng Q., Guo C., Zhao T., Wen Chen C. (2024). Target-Aware Camera Placement for Large-Scale Video Surveillance. IEEE Trans. Circuits Syst. Video Technol..

[5] Zhou M., Wei X., Wang S., Kwong S., Fong C.K., Wong P.H.W., Yuen W.Y.F. (2020). Global Rate-Distortion Optimization-Based Rate Control for HEVC HDR Coding. IEEE Trans. Circuits Syst. Video Technol..

[6] Zhou M., Zhang Y., Li B., Lin X. (2017). Complexity Correlation-Based CTU-Level Rate Control with Direction Selection for HEVC. ACM Trans. Multimed. Comput. Commun. Appl..

[7] Zhang W., Zhou M., Ji C., Sui X., Bai J. (2022). Cross-Frame Transformer-Based Spatio-Temporal Video Super-Resolution. IEEE Trans. Broadcast..

[8] Shen Y., Feng Y., Fang B., Zhou M., Kwong S., Qiang B.H. (2020). DSRPH: Deep semantic-aware ranking preserving hashing for efficient multi-label image retrieval. Inf. Sci..

[9] Gao T., Sheng W., Zhou M., Fang B., Luo F., Li J. (2020). Method for Fault Diagnosis of Temperature-Related MEMS Inertial Sensors by Combining Hilbert–Huang Transform and Deep Learning. Sensors.

[10] Wei X., Zhou M., Wang H., Yang H., Chen L., Kwong S. (2024). Recent Advances in Rate Control: From Optimization to Implementation and Beyond. IEEE Trans. Circuits Syst. Video Technol..

[11] Stępień I., Oszust M. (2025). Three-branch neural network for No-Reference Quality assessment of Pan-Sharpened Images. Eng. Appl. Artif. Intell..

[12] Tolie H.F., Ren J., Chen R., Zhao H., Elyan E. (2025). Blind sonar image quality assessment via machine learning: Leveraging micro- and macro-scale texture and contour features in the wavelet domain. Eng. Appl. Artif. Intell..

[13] Jingnan S., Mingliang Z., Luo J., Pu H., Yong F., Wei X., Weijia J. (2025). Boundary-Aware Feature Fusion with Dual-Stream Attention for Remote Sensing Small Object Detection. IEEE Trans. Geosci. Remote Sens..

[14] Cheng S., Song J., Zhou M., Wei X., Pu H., Luo J., Jia W. (2024). EF-DETR: A Lightweight Transformer-Based Object Detector with an Encoder-Free Neck. IEEE Trans. Ind. Inform..

[15] Zhou M., Zhao X., Luo F., Luo J., Pu H., Xiang T. (2023). Robust RGB-T Tracking via Adaptive Modality Weight Correlation Filters and Cross-modality Learning. ACM Trans. Multimed. Comput. Commun. Appl..

[16] Huang Y., Hechen Z., Zhou M., Li Z., Kwong S. (2025). An Attention-Locating Algorithm for Eliminating Background Effects in Fine-Grained Visual Classification. IEEE Trans. Circuits Syst. Video Technol..

[17] Zhou M., Wu X., Wei X., Xiang T., Fang B., Kwong S. (2024). Low-Light Enhancement Method Based on a Retinex Model for Structure Preservation. IEEE Trans. Multimed..

[18] Guo Q., Zhang Z., Zhou M., Yue H., Pu H., Luo J. (2023). Image Defogging Based on Regional Gradient Constrained Prior. ACM Trans. Multimed. Comput. Commun. Appl..

[19] Guo Q., Zhou M. (2022). Progressive Domain Translation Defogging Network for Real-World Fog Images. IEEE Trans. Broadcast..

[20] Mingliang Z., Shen W., Wei X., Luo J., Jia F., Zhuang X., Weijia J. (2025). Blind Image Quality Assessment: Exploring Content Fidelity Perceptibility via Quality Adversarial Learning. Int. J. Comput. Vis..

[21] Shen W., Zhou M., Wei X., Wang H., Fang B., Ji C., Zhuang X., Wang J., Luo J., Pu H. (2024). A Blind Video Quality Assessment Method via Spatiotemporal Pyramid Attention. IEEE Trans. Broadcast..

[22] Xian W., Zhou M., Fang B., Liao X., Ji C., Xiang T., Jia W. (2023). Spatiotemporal Feature Hierarchy-Based Blind Prediction of Natural Video Quality via Transfer Learning. IEEE Trans. Broadcast..

[23] Li B., Liang J., Fu H., Han J. ROI-Based Deep Image Compression with Swin Transformers. Proceedings of the ICASSP 2023—2023 IEEE International Conference on Acoustics, Speech and Signal Processing (ICASSP).

[24] Tirer T. Iteratively Preconditioned Guidance of Denoising (Diffusion) Models for Image Restoration. Proceedings of the ICASSP 2024—2024 IEEE International Conference on Acoustics, Speech and Signal Processing (ICASSP).

[25] Wang Z., Bovik A., Sheikh H., Simoncelli E. (2004). Image quality assessment: From error visibility to structural similarity. IEEE Trans. Image Process..

[26] Wang Z., Simoncelli E., Bovik A. Multiscale structural similarity for image quality assessment. Proceedings of the Thrity-Seventh Asilomar Conference on Signals, Systems Computers.

[27] Li C., Bovik A.C. Three-component weighted structural similarity index. Proceedings of the Image Quality and System Performance VI.

[28] Zhang L., Zhang L., Mou X., Zhang D. (2011). FSIM: A Feature Similarity Index for Image Quality Assessment. IEEE Trans. Image Process..

[29] Xue W., Zhang L., Mou X., Bovik A.C. (2014). Gradient Magnitude Similarity Deviation: A Highly Efficient Perceptual Image Quality Index. IEEE Trans. Image Process..

[30] Zhang L., Shen Y., Li H. (2014). VSI: A Visual Saliency-Induced Index for Perceptual Image Quality Assessment. IEEE Trans. Image Process..

[31] Narwaria M., Lin W. (2010). Objective Image Quality Assessment Based on Support Vector Regression. IEEE Trans. Neural Netw..

[32] Bosse S., Maniry D., Muller K.R., Wiegand T., Samek W. (2018). Deep Neural Networks for No-Reference and Full-Reference Image Quality Assessment. IEEE Trans. Image Process..

[33] Kim J., Lee S. Deep Learning of Human Visual Sensitivity in Image Quality Assessment Framework. Proceedings of the 2017 IEEE Conference on Computer Vision and Pattern Recognition (CVPR).

[34] Prashnani E., Cai H., Mostofi Y., Sen P. PieAPP: Perceptual Image-Error Assessment Through Pairwise Preference. Proceedings of the IEEE Conference on Computer Vision and Pattern Recognition (CVPR).

[35] Xian W., Zhou M., Fang B., Kwong S. (2022). A content-oriented no-reference perceptual video quality assessment method for computer graphics animation videos. Inf. Sci..

[36] Qiang B., Chen R., Zhou M., Pang Y., Zhai Y., Yang M. (2020). Convolutional Neural Networks-Based Object Detection Algorithm by Jointing Semantic Segmentation for Images. Sensors.

[37] Yan J., Zhang B., Zhou M., Campbell-Valois F.X., Siu S.W.I. (2023). A deep learning method for predicting the minimum inhibitory concentration of antimicrobial peptides against *Escherichia coli* using Multi-Branch-CNN and Attention. mSystems.

[38] Yan J., Zhang B., Zhou M., Kwok H.F., Siu S.W. (2022). Multi-Branch-CNN: Classification of ion channel interacting peptides using multi-branch convolutional neural network. Comput. Biol. Med..

[39] He K., Zhang X., Ren S., Sun J. Deep Residual Learning for Image Recognition. Proceedings of the 2016 IEEE Conference on Computer Vision and Pattern Recognition (CVPR).

[40] Zhang R., Isola P., Efros A.A., Shechtman E., Wang O. The Unreasonable Effectiveness of Deep Features as a Perceptual Metric. Proceedings of the IEEE Conference on Computer Vision and Pattern Recognition (CVPR).

[41] Liao X., Chen B., Zhu H., Wang S., Zhou M., Kwong S. DeepWSD: Projecting Degradations in Perceptual Space to Wasserstein Distance in Deep Feature Space. Proceedings of the 30th ACM International Conference on Multimedia.

[42] Liao X., Wei X., Zhou M., Wong H.S., Kwong S. (2025). Image Quality Assessment: Exploring Joint Degradation Effect of Deep Network Features Via Kernel Representation Similarity Analysis. IEEE Trans. Pattern Anal. Mach. Intell..

[43] Zhou M., Lan X., Wei X., Liao X., Mao Q., Li Y., Wu C., Xiang T., Fang B. (2023). An End-to-End Blind Image Quality Assessment Method Using a Recurrent Network and Self-Attention. IEEE Trans. Broadcast..

[44] Wei X., Li J., Zhou M., Wang X. (2022). Contrastive distortion-level learning-based no-reference image-quality assessment. Int. J. Intell. Syst..

[45] Duan H., Min X., Zhu Y., Zhai G., Yang X., Le Callet P. (2022). Confusing Image Quality Assessment: Toward Better Augmented Reality Experience. IEEE Trans. Image Process..

[46] Chen W., Cai B., Zheng S., Zhao T., Gu K. (2024). Perception-and-Cognition-Inspired Quality Assessment for Sonar Image Super-Resolution. IEEE Trans. Multimed..

[47] Zhou M., Leng H., Fang B., Xiang T., Wei X., Jia W. (2023). Low-light Image Enhancement via a Frequency-based Model with Structure and Texture Decomposition. ACM Trans. Multimed. Comput. Commun. Appl..

[48] Sheikh H., Bovik A. (2006). Image information and visual quality. IEEE Trans. Image Process..

[49] Chandler D.M., Hemami S.S. (2007). VSNR: A Wavelet-Based Visual Signal-to-Noise Ratio for Natural Images. IEEE Trans. Image Process..

[50] Bae S.H., Kim M. (2016). DCT-QM: A DCT-Based Quality Degradation Metric for Image Quality Optimization Problems. IEEE Trans. Image Process..

[51] Laparra V., Ballé J., Berardino A., Simoncelli E.P. (2016). Perceptual image quality assessment using a normalized Laplacian pyramid. Electron. Imaging.

[52] Gao F., Wang Y., Li P., Tan M., Yu J., Zhu Y. (2017). DeepSim: Deep similarity for image quality assessment. Neurocomputing.

[53] Ding K., Ma K., Wang S., Simoncelli E.P. (2022). Image Quality Assessment: Unifying Structure and Texture Similarity. IEEE Trans. Pattern Anal. Mach. Intell..

[54] Gu J., Cai H., Chen H., Ye X., Ren J., Dong C. (2020). Image quality assessment for perceptual image restoration: A new dataset, benchmark and metric. arXiv.

[55] Lao S., Gong Y., Shi S., Yang S., Wu T., Wang J., Xia W., Yang Y. Attentions Help CNNs See Better: Attention-based Hybrid Image Quality Assessment Network. Proceedings of the 2022 IEEE/CVF Conference on Computer Vision and Pattern Recognition Workshops (CVPRW).

[56] Shi W., Yang W., Liao Q. Robust Content-Variant Reference Image Quality Assessment Via Similar Patch Matching. Proceedings of the ICASSP 2023—2023 IEEE International Conference on Acoustics, Speech and Signal Processing (ICASSP).

[57] Chen C., Mo J., Hou J., Wu H., Liao L., Sun W., Yan Q., Lin W. (2024). TOPIQ: A Top-Down Approach From Semantics to Distortions for Image Quality Assessment. IEEE Trans. Image Process..

[58] Shen W., Zhou M., Luo J., Li Z., Kwong S. (2024). Graph-Represented Distribution Similarity Index for Full-Reference Image Quality Assessment. IEEE Trans. Image Process..

[59] Xian W., Zhou M., Fang B., Xiang T., Jia W., Chen B. (2024). Perceptual Quality Analysis in Deep Domains Using Structure Separation and High-Order Moments. IEEE Trans. Multimed..

[60] Shen W., Zhou M., Chen Y., Wei X., Luo J., Pu H., Jia W. Image Quality Assessment: Investigating Causal Perceptual Effects with Abductive Counterfactual Inference. Proceedings of the 2025 IEEE/CVF Conference on Computer Vision and Pattern Recognition (CVPR) (Accepted).

[61] Ding K., Ma K., Wang S., Simoncelli E.P. (2021). Comparison of Full-Reference Image Quality Models for Optimization of Image Processing Systems. Int. J. Comput. Vis..

[62] Wu T., Ma K., Liang J., Yang Y., Zhang L. A Comprehensive Study of Multimodal Large Language Models for Image Quality Assessment. Proceedings of the European Conference on Computer Vision (ECCV).

[63] You Z., Li Z., Gu J., Yin Z., Xue T., Dong C. Depicting Beyond Scores: Advancing Image Quality Assessment Through Multi-modal Language Models. Proceedings of the European Conference on Computer Vision (ECCV).

[64] Amirshahi S.A., Pedersen M., Stella X.Y. (2017). Image Quality Assessment by Comparing CNN Features between Images. Electron. Imaging.

[65] Fan L., Wei X., Zhou M., Yan J., Pu H., Luo J., Li Z. (2025). A Semantic-Aware Detail Adaptive Network for Image Enhancement. IEEE Trans. Circuits Syst. Video Technol..

[66] Gatys L.A., Ecker A.S., Bethge M. Image Style Transfer Using Convolutional Neural Networks. Proceedings of the 2016 IEEE Conference on Computer Vision and Pattern Recognition (CVPR).

[67] Johnson J., Alahi A., Fei-Fei L. Perceptual Losses for Real-Time Style Transfer and Super-Resolution. Proceedings of the the 14th European Conference on Computer Vision (ECCV).

[68] Sheikh H., Sabir M., Bovik A. (2006). A Statistical Evaluation of Recent Full Reference Image Quality Assessment Algorithms. IEEE Trans. Image Process..

[69] Larson E.C., Chandler D.M. (2010). Most apparent distortion: Full-reference image quality assessment and the role of strategy. J. Electron. Imaging.

[70] Ponomarenko N., Jin L., Ieremeiev O., Lukin V., Egiazarian K., Astola J., Vozel B., Chehdi K., Carli M., Battisti F. (2015). Image database TID2013: Peculiarities, results and perspectives. Signal Process. Image Commun..

[71] Lin H., Hosu V., Saupe D. KADID-10k: A Large-scale Artificially Distorted IQA Database. Proceedings of the Eleventh International Conference on Quality of Multimedia Experience (QoMEX).

[72] Gu J., Cai H., Chen H., Ye X., Ren J.S., Dong C. (2020). PIPAL: A Large-Scale Image Quality Assessment Dataset for Perceptual Image Restoration. Proceedings of the Computer Vision—ECCV 2020: 16th European Conference.

[73] Gottschalk P.G., Dunn J.R. (2005). The five-parameter logistic: A characterization and comparison with the four-parameter logistic. Anal. Biochem..

